# Bestrophin-Encoded Ca^2+^-Activated Cl^−^ Channels Underlie a Current with Properties Similar to the Native Current in the Moth *Spodoptera littoralis* Olfactory Receptor Neurons

**DOI:** 10.1371/journal.pone.0052691

**Published:** 2012-12-26

**Authors:** Adrien François, Marta Grauso, Elodie Demondion, Françoise Bozzolan, Stéphane Debernard, Philippe Lucas

**Affiliations:** 1 Institut National de la Recherche Agronomique, UMR 1272 Physiologie de l’Insecte : Signalisation et Communication, Versailles, France; 2 Université Pierre et Marie Curie, UMR 1272 Physiologie de l’Insecte: Signalisation et Communication, Paris, France; Albany Medical College, United States of America

## Abstract

Responses of insect olfactory receptor neurons (ORNs) involve an entry of Ca^2+^ through olfactory heterodimeric receptor complexes. In moths, the termination of ORN responses was found to strongly depend on the external Ca^2+^ concentration through the activation of unknown Ca^2+^-dependent Cl^−^ channels. We thus investigated the molecular identity of these Cl^−^ channels. There is compelling evidence that bestrophins form Cl^−^ channels when expressed in heterologous systems. Here we provide evidence that antennae of the moth *Spodoptera littoralis* express three transcripts encoding proteins with hallmarks of bestrophins. One of these transcripts, SlitBest1b, is expressed in ORNs. The heterologous expression of SlitBest1b protein in CHO-K1 cells yielded a Ca^2+^-activated Cl^−^ current that shares electrophysiological properties with the native Ca^2+^-activated Cl^−^ current of ORNs. Both currents are anionic, present similar dependence on the intracellular Ca^2+^ concentration, partly inactivate over time, have the same anion permeability sequence, the same sequence of inhibitory efficiency of blockers, the same almost linear *I–V* relationships and finally both currents do not depend on the cell volume. Therefore, our data suggest that SlitBest1b is a good candidate for being a molecular component of the olfactory Ca^2+^-activated Cl^−^ channel and is likely to constitute part of the insect olfactory transduction pathway. A different function (e.g. regulation of other proteins, maintenance of the anionic homeostasis in the sensillar lymph) and a different role (e.g. involvement in the olfactory system development) cannot be excluded however.

## Introduction

Olfaction is essential in guiding insect behaviors such as seeking mating partners and hosts, foraging, oviposition and avoidance of predators and lethal substances. This pivotal role of the olfactory system for survival and reproductive success is reflected in sophisticated olfactory structures and mechanisms [1], [2]. Olfactory receptor neurons (ORNs) are located within antennal cuticular structures called sensilla. The binding of odorant molecules to their cognate olfactory receptors (ORs) activates a signaling pathway transforming the olfactory stimulus in a graded electrical response, the receptor potential, and ultimately in a firing activity [3] that is processed in the antennal lobe [4].

ORNs face the challenge of converting the physical properties of the olfactory stimulus into trains of action potentials. Properties of an odor plume include not only the nature and intensity of the stimulus, but also its temporal pattern which is critical to elicit appropriate behaviors in insects, especially in the detection of the conspecific female sex pheromone by male moths [5]. The low quantity of pheromone emitted by calling females and the high velocity of flying insects impose strong constraints on the function of ORNs. Indeed, insect ORNs are extremely sensitive [6], fast [7], [8] and can resolve up to 10 short odor pulses per seconds [9]. Therefore, responses must contain sufficient information to encode both the onset and removal of a stimulus. This proves that these sensory neurons have a highly efficient transduction pathway.

By contrast to vertebrates, a clear complete model of the olfactory transduction is not yet available in insects and few molecular actors of the transduction cascade were identified [10], [11]. The question of whether insect ORs function like GPCRs or are modulated by G-proteins remains controversial [12]. Despite this uncertainty, it is clear that the activation of insect ORs leads to a Ca^2+^ entry in ORNs [13], [14]. The ensuing increase in the Ca^2+^ concentration shapes the electrical response of insect ORNs and is therefore crucial for encoding the intensitive and temporal characteristics of the stimulus. Indeed, lowering extracellular Ca^2+^ concentration delayed ORN repolarization [13]. We recently demonstrated in the Noctuid moth *Spodoptera littoralis* that Ca^2+^ activates a Cl^−^ current in ORNs [15]. *In vivo*, this Ca^2+^-activated Cl^−^ (CaC) current is involved in response termination and is therefore presumably required for ORN repolarization. The molecular identity of CaC channels in moth ORNs is unknown and their elucidation is an important step in understanding the precise role of these channels in insect olfactory transduction.

The identity of CaC channels remained obscure until recently. Of the existing candidates, two families recapitulate reliably the properties of CaC channels, bestrophins [16] and anoctamins (also named TMEM16) [17]. In vertebrates, the response of canonical ORNs [18] and vomeronasal neurons [19] includes the opening of CaC channels. Vertebrate ORNs express a member of the anoctamin family, anoctamin 2 (Ano2), in their cilia [20], [21]. In mice, ablation of the *Ano2* gene proved that it is the major, or perhaps the only, subunit of the CaC current in the cilia of ORNs and in vomeronasal neurons, although the importance of this channel for odor perception has been questioned [22]. Cilia of vertebrate ORNs also express a member of the bestrophin family, bestrophin-2 (Best2), where it colocalizes with the channel responsible for the primary transduction current [23]. However, the role of Best2 remains obscure as Best2 disruption did not modify CaC currents [24].

The founding member of bestrophins, human bestrophin-1 (hBest1), encoded by the *VMD2* gene, was identified as the gene responsible for Best macular dystrophy, a degeneration of the retinal pigment epithelium [25], [26]. Three or four bestrophin genes are known in different mammalian species and they are described as widely expressed plasma membrane channels involved in various functions [27]. They are generally believed to form CaC channels [28]–[38] and/or to regulate ion channels [39]–[41]. hBest1 was recently found to be involved in Ca^2+^ handling in endoplasmic reticulum stores [42], [43]. Moreover some bestrophins are volume-regulated anion channels (VRACs) [44], [45] and could be involved in cell-volume regulation and Cl^−^ homeostasis [46], [47]. Bestrophin homologues were identified in several invertebrates from public databases [48]. To our knowledge, the function of insect bestrophins has only been studied in *Drosophila*. As their vertebrate counterparts, *Drosophila* bestrophins form CaC current in expression systems [32] and in native S2 cells [28].We describe here the molecular characterization of bestrophin cDNAs isolated from *S. littoralis* antennae and show that one bestrophin, SlitBest1b, is expressed in ORNs. We characterized its functional properties in a heterologous expression system. The similar electrophysiological and pharmacological properties of the recombinant SlitBest1b current to the native current suggest that SlitBest1b is a good candidate for being a molecular component of the CaC channels identified in moth ORNs.

## Materials and Methods

### Insects

*Spodoptera littoralis* moths (Lepidoptera, Noctuidae) were reared in our laboratory at 23°C and fed on an artificial diet. Pupae were sexed and males and females kept separately. Three-day-old male pupae were selected for primary cell cultures. Tissues from adults (antennae, brains, proboscises, legs, thoraces, abdomens and wings) were dissected and used directly for total RNA isolation. For the developmental study, antennae were collected from pupae (day 7 and 11) and adults (days 1 to 4) and used for RNA isolation.

### RNA Isolation and cDNA Synthesis

Total RNAs were extracted with TRIzol reagent (Invitrogen, Carlsbad, CA, USA), then treated with DNase I (Roche, Basel, Switzerland), according to the manufacturer’s instructions and were quantified by spectrophotometry at 260 nm. Single-stranded cDNAs were synthesized from total RNAs (5 µg) from various tissues using Superscript II reverse transcriptase (Invitrogen) with the oligo(dT)_18_ primer according to the manufacturer’s instructions. For 5′- and 3′-rapid amplification of cDNA ends (RACE) PCR, cDNA was synthesized from 1 µg of male antennal total RNA using the SMART RACE cDNA Amplification Kit (Clontech, Mountain View, CA, USA).

### Cloning of Bestrophins

Sequence similarity search on a *S. littoralis* male antenna EST library [49] was conducted by BLAST [50] with the sequence of the three mouse bestrophins [51] and the four *Drosophila* bestrophins (Genbank accession numbers: AAF54503.1, AAF50668.2, AAF49648.1, AAF49649.3). Three clones exhibiting the highest similarity scores were recovered from the library, fully sequenced and named SlitBest1a, SlitBest1b and SlitBest2. For protein analysis the following tools were run: ClustalW2 (http://www.ebi.ac.uk/Tools/clustalw2/) for sequence alignment, BoxShade (http://mobyle.pasteur.fr/cgi-bin/portal.py?) for alignment drawing, the TMHMM v. 2.0 server (http://www.cbs.dtu.dk/services/TMHMM/) for transmembrane helice prediction, the NetPhosK 1.0 server (http://www.cbs.dtu.dk/services/NetPhosK/) for kinase-specific phosphorylation site prediction.

### Expression Analysis

Non-quantitative RT-PCR was performed on 100 ng of cDNAs from different tissues by using specific primers for each *S. littoralis* bestrophin and the control gene RpL13 whose expression was previously shown not to vary with the age of males [52]. Thirty-five cycles of amplification were realized for bestrophins and 30 cycles for RpL13 in order to fit the linear range of amplification.

For developmental analysis of SlitBest1b expression by qPCR, all reactions were performed on the LightCyclerH 480 Real-Time PCR System (Roche). The reference genes (RpL13, RpL8, GAPDH and β-actin) and their corresponding primers were previously described [52]. Each 12-µl reaction consisted in 6 µl LightCyclerH 480 SYBR Green I Master (Roche), 4 µl of 10-fold diluted cDNA (or water for negative control) and 0.6 µl of each primer. The qPCR program was 95°C for 5 min, then 45 cycles of 95°C for 10 s, 60°C for 15 s, 72°C for 15 s. A fivefold dilution series were used to construct a relative standard curve to determine the PCR efficiencies (90–100%). Each reaction was run in triplicate on independent biological samples. Data were analysed with LightCycler 480H Software (Roche) and the crossing point values (Cpvalues) were first determined for the reference genes. The RpL13 gene was considered as displaying steady expression and was suitable for downstream analysis, as previously described [52]. Subsequently, the expression of SlitBest1b was normalized to geometric means of this reference and the normalized gene expression was then calculated with Q-Gene software [53].

### ORNs Primary Culture

Primary cultures of male ORNs were prepared as previously described [54]. Antennal flagella from 3-days-old male pupae were dissected. After mechanical and enzymatic dissociations, cells were plated onto uncoated Falcon Petri dishes. The culture medium consisted in three parts of Leibovitz L15 medium, two parts of Grace medium conditioned on the embryonic cell line MRRL-CH1 and 5% of fetal bovine serum (Invitrogen). Cultures were maintained in an incubator at 20°C. The culture medium was changed every 7 days.

### Single-Cell RT-PCR

The single-cell RT-PCR protocol was modified from [55]. Patch-clamp recordings were performed from 10-to-15-day-old cultures as previously described [15]. In the whole-cell configuration, the cytosol of ORNs exhibiting a CaC current was harvested within the pipette filled of RNase-free solution containing (in mM) 140 CsCl, 1 CaCl_2_, 2 MgCl_2_, and 10 HEPES (pH 7.4, 325 mosmol/L). Tubes were immediately put in liquid nitrogen and stored at −80°C before use.

Three-to-four collected cytosols were used for RT with RNAse inhibitor (40 U, 1 µl, Promega), RQ1 DNAse (1 U, 1 µl, Promega) and 5× MMLV Buffer (4 µl, Clontech) and the mix was incubated 30 min at 37°C and 10 min at 65°C. Then 20 µM Random primers, Oligo dTs (1 µl each, Clontech), 10 mM dNTPs Mix (1 µl, Clontech) and MMLV Reverse Transcriptase (200 U, 1 µl, Clontech) were added and the final 20 µl-solution was successively incubated 10 min at 25°C, 50 min at 42°C and 15 min at 70°C.

A multiplex PCR (PCR1) was first carried out for the simultaneous amplification of *S. littoralis* bestrophins and two control genes (RpL8 and SlitOrco). Sixty microliters of a PCR mix containing 10 mM dNTPs Mix (2 µl), 2 µl 50× Titanium *Taq* DNA polymerase and 10 µl of 10× PCR buffer (Clontech) were added to the RT product with 20 µl of a mix containing the sense and antisense primers (10 pmol each) (SlitBest1a-SC.F/SlitBest1-SC.R1, SlitBest1b-SC.F/SlitBest1-SC.R1, SlitBest2-SC.F/SlitBest2-SC.R1, SlitOrco-SC.F/SlitOrco-SC.R1, RpL8-SC.F/RpL8-SC.R1 primers are shown in Table 1). After 1 min at 94°C, samples were processed for 35 cycles of 95°C for 30 s, 60°C for 30 s, and 68°C for 1 min. Then, a nested PCR was performed on 2 µl of PCR1 products with 46 µl of a reaction mix containing 5 µl 10 X Titanium *Taq* buffer, 1 µl 50 X Titanium *Taq* DNA polymerase, and 1 µl of dNTPs (10 mM) and an antisense primer specific to each gene (SlitBest1-SC.R2, SlitBest2-SC.R2, SlitOrco-SC.R2 and RpL8-SC.R2). The samples were processed as described above and subjected to 40 additional PCR cycles.

**Table 1:** List of the primers used for single-cell reverse transcription polymerase chain reaction (RT-PCR).

Sense primer		Antisense primer	
**SlBest1a-SC.F**	5′-CCGTGGCGTGAAACTGAAGAAA (PWRETEE)	**SlBest1-SC.R1**	5′-ACTCTTGGTGAGATCATCGTGAGC (LTMISPR)
**SlBest1b-SC.F**	5′-CGAGGTAGCAACTTGTCGTGGTTT (EVATCRG)	**SlBest1-SC.R2**	5′-GTAAGGCAGAGACACACGTAGCGG (RYVCLCL)
**SlBest2-SC.F**	5′-CGGCAGCAGTTTCGGATGTTTCTG (GSSFGCF)	**SlBest2-SC.R1**	5′-CGACCCGTCTCGTCTTTACTAACC (VSKDETG)
		**SlBest2-SC.R2**	5′-ATGTTGCCACGTTGGAAACCTTCG (RRFPTWQH)
**SlOrco-SC.F**	5′-CTTTATCTGCGGCATGACTGTCCT	**SlOrco-SC.R1**	5′-ACTGCACCAAGTACCGAAGCAA
		**SlOrco-SC.R2**	5′-TCGGAGTCAAGCCATTAGGGTTGT
**RpL8-SC.F**	5′-GTGATTCGTGCTCAGCGTAAAGGT	**RpL8-SC.R1**	5′-TGAGGATGCTCAACGGGGTTCATA
		**RpL8-SC.R2**	5′-CCAATGACAGTGGCGAAGTTTCCT

Corresponding amino acid sequences are indicated between brackets.

### CHO-K1 Transfection

The full open reading frame of SlitBest1b, without the stop codon, was cloned in the pCINeo/IRES-GFP bicistronic mammalian expression vector [56]. Chinese hamster ovary (CHO-K1) cells were cultured at 37°C and 5% CO_2_ in Dulbecco’s modified Eagle’s medium (DMEM, Sigma) supplemented with 10% heat inactivated fetal bovine serum (Fischer). About 10^5^ cells were plated in 35 mm Petri dishes 24 hours before transient transfection with 1 µg of the SlitBest1b expression plasmid using Lipofectamine 2000 and OptiMEM (Invitrogen).

### Whole-cell Recordings

SlitBest1b currents were recorded in the whole-cell patch-clamp configuration from green fluorescent protein (GFP) expressing CHO-K1 cells 24 to 48 hours after transfection. Electrodes pulled from borosilicate capillaries (GC150-10, Harvard Apparatus, Les Ulis, France) by a Sutter P-97 puller (Sutter Instrument Co., Novato, CA, USA) had resistances of 6–10 MΩ when filled with the standard intracellular solution. An Ag-AgCl reference electrode was connected to the bath through an agar bridge filled with the pipette solution. Currents were recorded with an Axopatch 200 B and digitized at 20 kHz using a Digidata 1322A (Molecular Devices). During whole-cell recordings, the membrane potential was clamped to −60 mV. Data were acquired and analyzed with pClamp 10 (Molecular Devices). We measured both the peak amplitude of inward currents and the mean amplitude of steady-state currents calculated between 150 and 250 s after breaking into the whole-cell configuration. Current–voltage (*I–V*) relationships were generated from voltage step protocols or ramp protocols. To subtract leak currents, currents recorded with a voltage protocol generated within the first 5 s of whole-cell mode were subtracted from currents recorded during subsequent protocols. Cell capacitance, determined from the capacitive current elicited by a 10 mV depolarizing voltage step ranged from 2 to 8.6 pF (4.2±0.13 pF; *n = *112). Currents are expressed as density in pA/pF.

### Solutions and Drugs

For whole-cell recordings, the standard intracellular pipette solution had the following composition (in mM): 122 N-methyl-D-glucamine (NMDG)-Cl, 1.1 CaCl_2_, 11 EGTA, 10 HEPES, pH 7.2, adjusted to 290 mosmol/L with mannitol. The free Ca^2+^ concentration was 20 nM as calculated with WebmaxC v.2.20. SlitBest1b currents were activated with different free Ca^2+^ concentrations in the pipette adjusted according to Table 2. The standard extracellular bath solution (in mM): 123 Na-gluconate, 1 CaCl_2_, 5 glucose, and 10 HEPES, pH 7.2, adjusted to 300 mosmol/L with mannitol. This low-Cl^−^ bath solution was used to generate inwardly rectified Cl^−^ currents easy to discriminate from possible leak currents.

**Table 2:** Compositions of pipette solutions for dose-response experiments.

Free-Ca^2+^ (M)	NMDG-Cl (mM)	CaCl_2_ (mM)	EGTA (mM)	HEDTA (mM)	NTA (mM)
2×10^−8^	122.8	1.1	11	–	–
1×10^−7^	117.8	3.6	10	–	–
1×10^−6^	122.4	1.3	–	10	–
1×10^−5^	113	6	–	10	–
1×10^−4^	121.4	1.8	–	–	5
1×10^−3^	123	1	–	–	–

All solutions contained 10 mM HEPES. The pH was adjusted to 7.2 with NMDGOH and osmotic pressure was 290 mosmol/L. Free Ca^2+^ concentrations were calculated with WebmaxC Standard (http://www.stanford.edu/~cpatton/webmaxc/webmaxcS.htm).

Blockers of the CaCl current, flufenamic acid, 5-nitro-2-(3-phenylpropylamino) benzoic acid (NPPB), and niflumic acid, were dissolved in dimethyl sulfoxide (DMSO). All stock aliquots were stored at −20°C. For drug application, the final DMSO concentration was ≤0.1%. This concentration of solvent had no effect on electrophysiological properties of ORNs. All drugs and chemicals were purchased from Sigma-Aldrich (Saint-Quentin Fallavier, France).

### Statistical Analyses

All results are expressed as means ± SEM. The nonparametric Mann–Whitney two-tailed test (for pharmacological analysis), a one-way ANOVA followed by Dunnett’s multiple-comparison test (for relative anion permeability and conductance experiments) and a one-way ANOVA followed by Turkey test (for qPCR) were used to determine statistical significance of differences between groups.

## Results

### Cloning and Sequence Analysis of Three *S. littoralis* Bestrophins

In order to reveal the molecular identity of the channel underlying the CaC current that we previously described in moth ORNs [15], we screened by BLAST a *S. littoralis* male antenna EST library. We found several EST fragments (Genbank accession numbers: FQ031133.1, FQ021050.1, FQ028240.1, FQ014676.1, FQ020755.1, FQ022393.1 and FQ017788.1) sharing high similarity with the bestrophin’s Cl^−^ channel family. The full sequencing of these EST clones showed that they represented three different full-length cDNAs probably derived from the expression of two distinct genes, one of them giving two variants differing in their 5′ UTR and NH3-terminal portion. We named these cDNAs SlitBest1a, SlitBest1b and SlitBest2 (Genbank accession numbers are JQ968533, JQ968534, JQ968535 respectively) because the encoded proteins present 48%, 51%, 36% and 31%, 33%, 62% of identity with the *Drosophila* Best1 and Best2 proteins, respectively.

**Figure 1:**
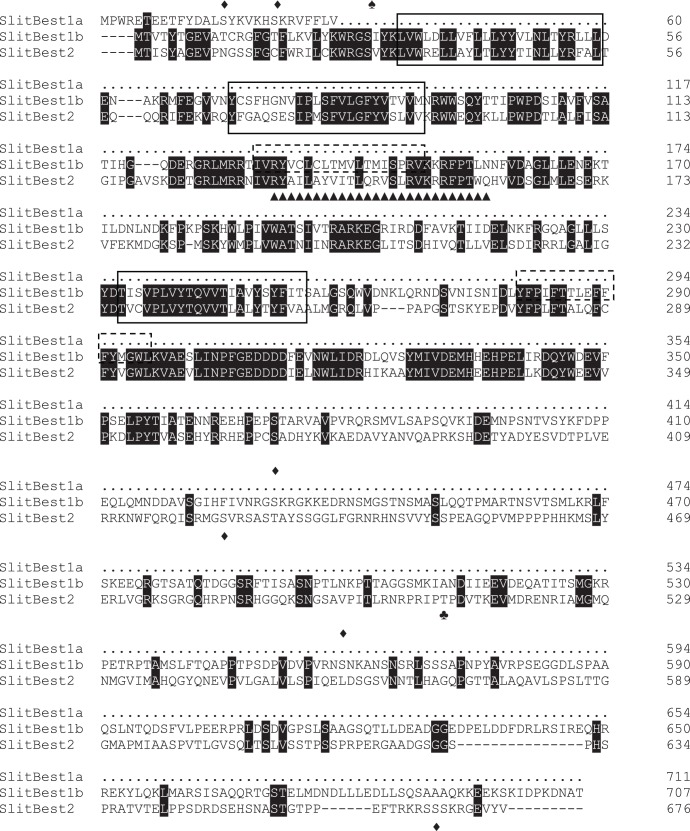
Alignment of *S. littoralis* bestrophins proteins. Alignment was realized with ClustalW2 (http://www.ebi.ac.uk/Tools/clustalw2/) and displayed with BoxShade (http://mobyle.pasteur.fr/cgi-bin/portal.py?) The amino acid sequence of SlitBest1a that matches with SlitBest1b is omitted. Identical amino acids between all sequences are marked in black. Predicted transmembrane domains are surrounded by a solid line for domains shared by the three sequences and by a dotted line for the Slitbest1a and SlitBest1b transmembrane domains. Predicted PKC, PKA and PKB phosphorylation sites are indicated by diamonds (♦), spades (♠) and clubs (♣), respectively. The bestrophin RFP domain is indicated by triangle (▴).

*In silico* analysis of the *S. littoralis* bestrophin proteins identified the following features: (1) five predicted transmembrane domains in SlitBest1 but only three in SlitBest2; (2) conservation of RFP domain that is believed to specify the ionic selectivity of the pore channel [33]; (3) some kinase-specific phosphorylation sites are predicted with an high (more than 0.8) probability score (Figure 1).

### Tissue-related Expression of *S. littoralis* Bestrophins

**Figure 2:**
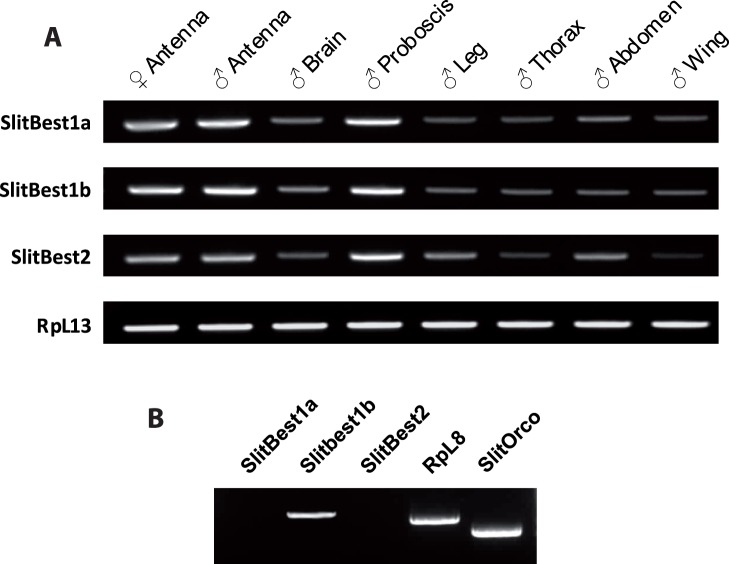
Tissue expression of *S. littoralis* bestrophins. A ) Analysis of expression of the three *S. littoralis* bestrophins in various adult tissues by RT-PCR showing that transcript levels are higher in chemosensory organs (antennae and proboscis), with no differences between male and female antennae. RpL13 was used as control gene. **B**) Single-cell RT-PCR from ORNs in primary culture. Among the three bestrophins, only SlitBest1b transcript was detected along with the control genes SlitOrco and RpL8.

The tissue distribution of *S. littoralis* bestrophins was analyzed by RT-PCR on total RNA samples extracted from 1-day-old males by using a pair of specific DNA primers for each transcript. The ubiquitous ribosomal gene RpL8 was used as positive PCR control. The RT-PCR analysis revealed the amplification of SlitBest1a, SlitBest1b and SlitBest2 cDNA fragments of expected size (241 bp, 212 bp and 194 bp) in all tested tissues and a high expression level for all the three bestrophins was detected in the antenna (male and female) and proboscis (Figure 2A).

To precise the expression of SlitBest1a, SlitBest1b and SlitBest2 within olfactory sensilla, single-cell RT-PCR experiments were performed from cultured ORNs. For RT-PCR positive controls, ORN cDNAs were used as template to amplify the housekeeping RpL8 gene and the obligate olfactory co-receptor SlitOrco. Single cell RT-PCRs revealed a transcriptional activity of SlitBest1b, SlitOrco and RpL8 genes in ORNs whereas SlitBest1a and SlitBest2 were not detected (Figure 2B). None of these genes were amplified from the culture medium (data not shown).

### Developmental Expression of SlitBest1b

**Figure 3:**
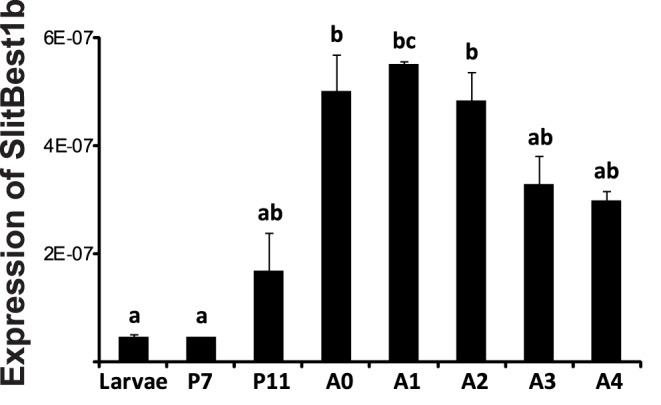
Developmental analysis of SlitBest1b expression in male antennae. SlitBest1b expression was investigated by qPCR on larvae (last instar stage), pupae (7 and 11 days after pupation, P7 and P11) and adults (the day of adult emergence (A0) and the next 4 days, A1 to A4). The transcript level increased from day 7 to day 11 of pupation and reached a peak at emergence that was maintained during the first three days of the adult stage and then it slightly decreased. RpL13 gene was used for normalization. Data were obtained from triplicate experiments and are given as means ± SD. Bars with same letters are not significantly different (ANOVA; Tukey test; p<0.05).

The expression level of SlitBest1b was quantified by real-time PCR in male antennae at different ages. The SlitBest1b transcript was weakly expressed in last instar larvae and at the middle of the pupal stage (P7) (Figure 3). The level of transcript increased at the end of the pupal stage (P11) to reach a maximum at the time of adult emergence and remained close to this level during the two following days of the adult stage and then slightly decreased (Figure 3).

### SlitBest1b Induces a Ca^2+^-dependent Cl^−^ Current in CHO-K1 cells

**Figure 4:**
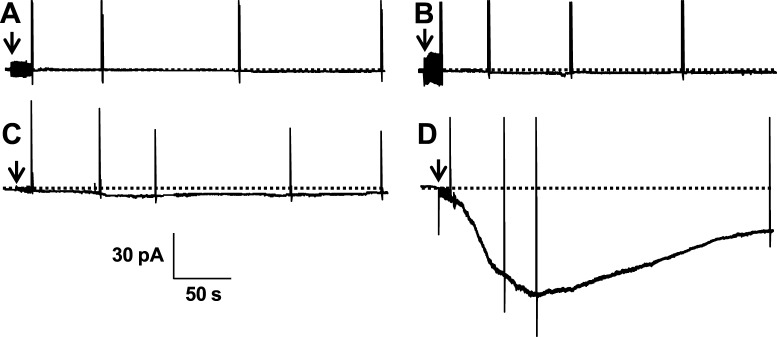
Currents elicited by SlitBest1b in CHO transfected cells. No currents were recorded with 100 µM intracellular Ca^2+^ from non-transfected cells (**A**) and from cells transfected only with GFP (**B**). CHO-K1 cells transfected with SlitBest1b and GFP did not exhibit any current when recorded with a 20 nM intracellular Ca^2+^ solution (**C**) whereas with a 100 µM intracellular Ca^2+^ solution an inward current was elicited (**D**). Recording were made at −60 mV holding potential. Arrows indicate the transition from cell-attached to whole-cell configuration. Dotted horizontal lines are zero current levels.

Functional properties of SlitBest1b were analyzed with whole-cell patch-clamp recordings from CHO-K1 cells transiently cotransfected with cDNAs for GFP and SlitBest1b. No currents were observed at −60 mV holding potential from non-transfected CHO-K1 cells recorded with 20 nM (*n* = 4) or 100 µM (*n* = 7) intracellular Ca^2+^ concentrations (Figure 4A). Control GFP-transfected cells also did not show any current at 100 µM intracellular Ca^2+^ (Figure 4B) as well as GFP/SlitBest1b transfected cells recorded with 20 nM intracellular Ca^2+^ (Figure 4C). By contrast, 87% of GFP/SlitBest1b cells recorded with at least 1 µM intracellular Ca^2+^ (n = 75) exhibited an inward current that slowly developed after breaking into the whole-cell configuration. This current reached a peak within 30–150 s, exhibited a marked decrease in amplitude and attained a steady state (Figure 4D). The mean amplitude of the steady-state current, measured between 150 and 280 s after breaking in whole-cell, was 58±6% (*n = *21) of the peak current elicited with 10 µM or 100 µM internal Ca^2+^ (Figure 4D).

**Figure 5:**
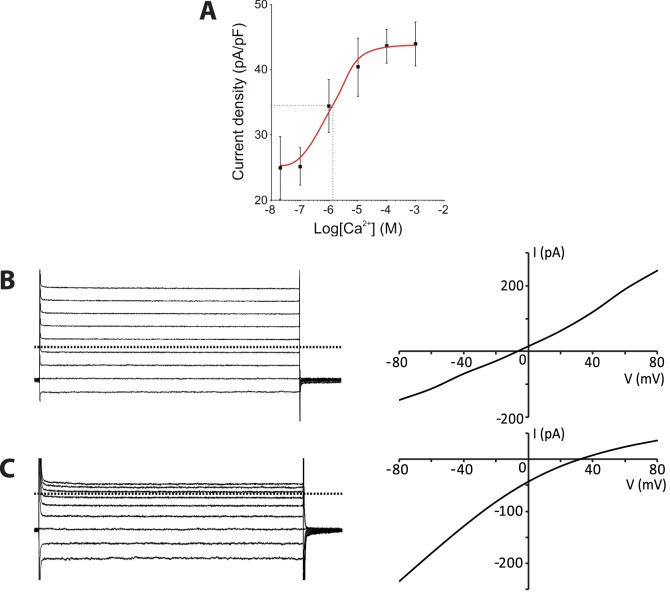
The SlitBest1b current depends on the Ca^2+^ concentration and is anionic. A ) Dose-response recordings were conducted in a low-Cl^−^ external solution. The curve fitted to the Hill equation has an EC_50_ of 1.2 µM and a Hill coefficient of 1.0. Means ± SEM. Representative currents recorded during voltage step protocols and corresponding *I-V* relationships from SlitBest1b transfected cells dialyzed with 100 µM intracellular Ca^2+^, **B**) in symmetrical Cl^−^ concentrations (*E*_Cl_ = 0 mV), and **C**) in low-Cl^−^ bath solution (*E*_Cl = _104 mV). Dotted horizontal lines are zero current levels.

We then established the relationship between the amplitude of the current in SlitBest1b transfected cells and the intracellular Ca^2+^ concentration from 20 nM to 1 mM. The maximal current density was reached at ca. 100 µM Ca^2+^ (Figure 5A). Fitting of the data to the Hill equation yielded a half-maximum current density (EC_50_) at the Ca^2+^ concentration of 1.2 µM and a Hill coefficient of 1.0.

To determine the channel selectivity we compared the *I–V* relationships and the reversal potentials of Ca^2+^-activated currents in different extracellular ionic conditions. In symmetrical Cl^−^ concentrations, the *I–V* relationship of the current activated with 100 µM intracellular Ca^2+^ was almost linear (Figure 5B) and the mean reversal potential was −12.8±3.9 mV (*n = *4). The reversal potential of the SlitBest1b current showed no dependence on extracellular Na^+^ concentration (−11.6±4.0 mV, *n* = 4). In contrast, after the exchange of all but 2 mM of extracellular Cl^−^ with the impermeable anion gluconate^−^ (*E*_Cl_ = 104 mV), the Ca^2+^-activated current became inwardly rectified (Figure 5C) and the reversal potential shifted to positive values (43.3±14.9 mV, *n = *23), indicating that Ca^2+^ activates an anionic current in GFP/SlitBest1b CHO-K1 cells.

To establish whether the SlitBest1b current depends on cell volume, cells were bathed with a solution adjusted to 300 mosmol/L and recorded with 100 µM Ca^2+^ pipette solutions adjusted to an osmotic pressure of 290 mosmol/L (hyperosmotic condition) or to 330 mosmol/L (hypoosmotic condition). Currents activated in hyperosmotic or hyposmotic conditions did not differ significantly in their *I-V* relationship and presented similar reversal potentials (−12.8±3.9 mV *vs.* −14.2±4.3 mV). Maximal current densities (43.1±4.7 pA/pF *vs*. 43.6±5.9 pA/pF; n = 4), time to reach the maximal current density (67.2±9.4 s *vs*. 63.2±11.2 s; n = 4), and percentage of sustained current (54.6±12.3% *vs*. 56.3±9.8%; n = 4) were also comparable, indicating that SlitBest1b currents are not cell-volume dependent.

### Relative Permeability, Conductance and Inhibitors of Ca^2+^-activated Channels

The anion relative permeability and conductance of channels responsible for the recombinant Ca^2+^-activated current of GFP/SlitBest1b CHO-K1 cells were determined by substituting extracellular Cl^−^ with equimolar quantities of bromide, iodide, nitrate, methanesulphonate, or gluconate. Currents were activated by dialysis with 100 µM Ca^2+^ and recorded in 125 mM internal Cl^−^.

**Figure 6:**
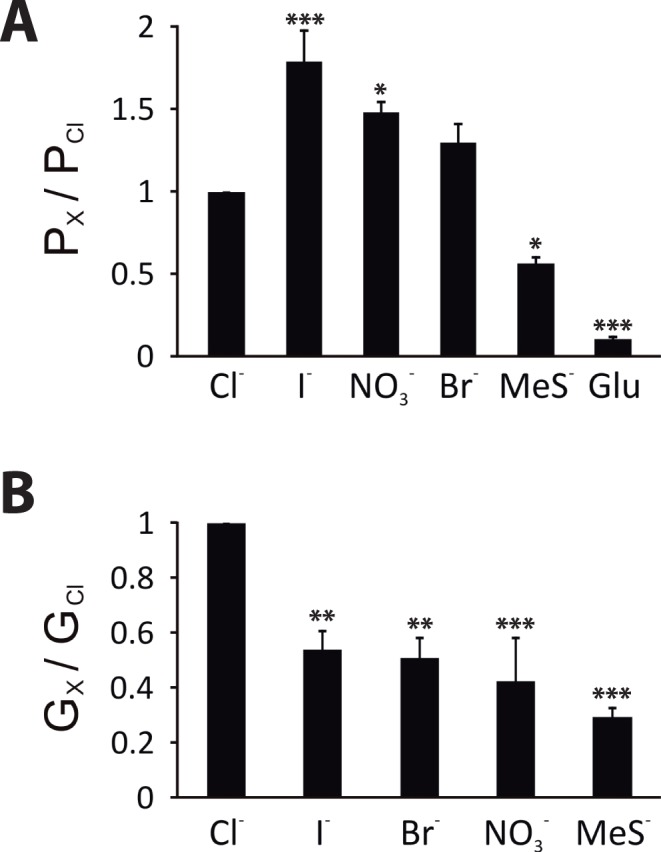
Anion relative permeability and relative slope conductance of the SlitBest1b current. Whole-cell currents were activated by dialysis with 10 µM free Ca^2+^ and recorded in bath solutions containing either 125 mM NaCl or 125 mM of the substituting NaX. **A**) Anion relative permeabilities *P*_X_/*P*_Cl_ were calculated using the Goldman–Hodgkin–Katz equation from measured differences in *E*_rev_ between symmetrical Cl^−^ and bianionic conditions. **B**) Relative slope conductances *G*_X_/*G*_Cl_ were obtained from the measurement of the slope of the *I-V* relationships between −80 and +80 mV. Four replicates for each condition were obtained. Means ± SEM. *p<0.05; **p<0.01; ***p<0.001.

Permeability ratios relative to Cl^−^ (*P*_X_ */P*_Cl_, where X indicates the substituting anion) were estimated by the shift in the reversal potential of the current under extracellular bianionic conditions and were calculated using the Goldman–Hodgkin–Katz equation as follows: *P_X_/P*_Cl_ = [Cl]_i_/{[X]_o_ exp(Δ*E*_rev_ *F*/*RT*)}−[Cl]_o_/[X]_o_ with Δ*E*_rev_ = *E*_X_−*E*_Cl_, where *E*_X_ is the reversal potential of the current in bianionic conditions, *F* is the Faraday constant, *R* is the gas constant and *T* is the absolute temperature. *E*_rev_ values measured after subtraction of leak currents were −12.8±3.9 mV for Cl^−^ (*n = *4), −18.6±4.8 mV for Br^−^ (*n = *4), −22.1±2.4 mV for NO_3_^−^ (*n = *3), −28±4.8 mV for I^−^ (*n = *4), 2.3±3.7 mV for CH_3_SO_3_^−^ (*n = *4), and 43.3±14.9 mV for gluconate^−^ (*n = *23). Thus, the anion permeability sequence was I^−^>NO_3_^−^>Br^−^>Cl^−^>CH_3_SO_3_^−^>>gluconate^−^, and the relative permeability ratios were I^−^:NO_3_^−^:Br^−^:Cl^−^:CH_3_SO_3_^−^:gluconate^−^ = 1.8∶1.5∶1.3∶1:0.6∶0.1 (Figure 6A). To calculate the relative conductance of substituting anions versus Cl^−^ (*G_X_*/*G*_Cl_) we measured the slope of each *I–V* relationship between −80 and +80 mV after subtraction of leak currents. The relative conductance ratios were NO_3_^−^:Br^−^:Cl^−^:CH_3_SO_3_^−^ = 0.5∶0.5∶0.4∶0.3 (Figure 6B). All tested permeant anions produced a significantly lower conductance than Cl^−^.

**Figure 7:**
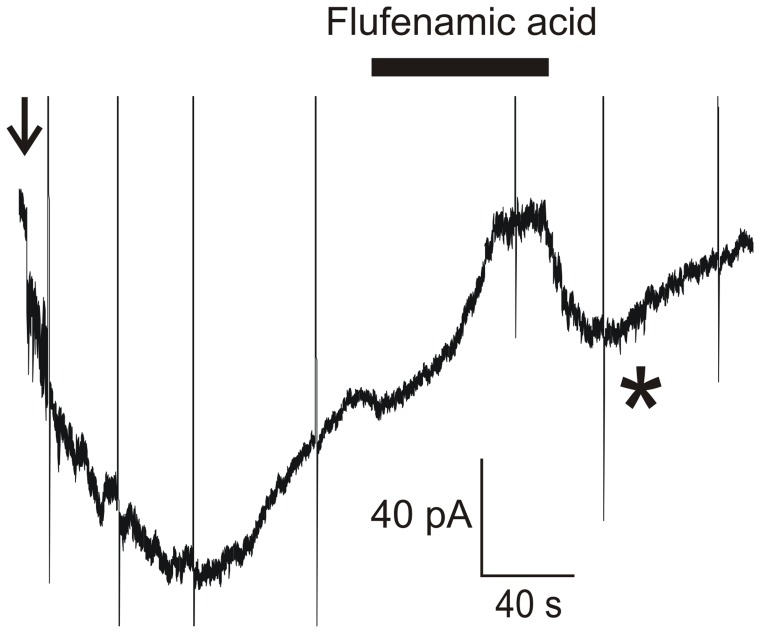
Cl^−^ channel inhibitors reversibly block the SlitBest1b current. **A**) Application of 100 µM flufenamic acid on the steady state of the whole-cell Ca^2+^-activated current recorded at a holding potential of −60 mV. Recordings were done in low-Cl^−^ external solution. Currents recorded during voltage protocols are truncated. The star indicates the transient current rebound observed after washing out flufenamic acid. The arrow indicates the transition from cell-attached to whole-cell configuration.

We investigated the pharmacological profile of the CaC current by testing three blockers, NPPB (100 µM), flufenamic acid (100 µM), and niflumic acid (300 µM), that were described as inhibiting CaC currents in vertebrates [57]. Drugs were applied on the steady-state of the current activated by 100 µM intracellular Ca^2+^ and recorded at a holding potential of −60 mV. Bath application of any of the three Cl^−^ channel blockers reversibly inhibited the Ca^2+^-activated current (Figure 7). A transient rebound of the current was observed upon removal of the drug and then the current amplitude decreased again. The sequence of inhibitory efficiency of the CaC current was NPPB>flufenamic acid>niflumic acid (Table 3).

**Table 3:** Summary of effects of three CaC channel inhibitors tested on SlitBest1b currents.

Inhibitors	Concentration	% Inhibition	% Recovery at the peak
NPPB	100 µM	101.1±7.6 (n = 5)	108.9±21.1 (n = 4)
Flufenamic acid	100 µM	78.7±8.2 (n = 4)	92.6±23.2 (n = 4)
Niflumic acid	300 µM	65.1±8.9 (n = 4)	81.7±11.4 (n = 4)

Mean ± sem.

## Discussion

The response of insect ORNs involves the sequential opening of Ca^2+^-permeable channels [58], [59], and CaC channels [15]. The data presented in this study reveal that antennae of *S. littoralis* express three transcripts encoding bestrophins. One of these transcripts, SlitBest1b, is expressed in ORNs. Heterologous expression of SlitBest1b in CHO-K1 cells yielded a CaC current that shares electrophysiological properties with the native CaC current expressed by ORNs.

### Primary Structure of *S. littoralis* Bestrophins

Although different models are proposed for the membrane topology of bestrophins, it is admitted that these proteins are highly conserved in the N-terminal region and only differ in their C-terminus [27]. Thus, a high conservation was observed in the first 355 amino acids of the SlitBest1a, SlitBest1b and SlitBest2, a region that includes all the features of the bestrophin family: several adjacent transmembrane domains (five predicted for SlitBest1a, SlitBest1b and three for SlitBest2), a conserved RFP domain spanning the putative third transmembrane segment of SlitBest1, and multiple protein kinase phosphorylation sites.

The second transmembrane domain, supposed to be involved in the constitution of the pore of the channel, is one of the most conserved sequences between the three *S. littoralis* bestrophins and bestrophins from other phyla (not shown). Its role in forming the channel pore is supported by the observation that deletions of many of the residues within this region altered the relative conductance and permeability of the channel to anions [33]. The bestrophin RFP region has been originally identified by its similarity to the second transmembrane domain (M2) of ligand-gated anion channels belonging to the GABA_A_ and glycine receptor family. Moreover, the RFP domain was showed to have similar biophysical properties to M2 in terms of anionic selectivity and estimated pore dimensions and orientation [33]. Site directed mutagenesis experiments provided evidence that the ionic selectivity required the formation of rings by the RFP domain with the contribution of three conserved residues, two arginines and one proline that are present in SlitBest sequences.

As in all bestrophins from nematodes to mammals, different phosphorylation sites for PKC, PKA and PKB were predicted for the *Spodoptera* bestrophins. The role of kinase sites has not been yet well established for bestrophins. The only evidence that bestrophins are modulated by phosphorylation came from one study showing that in human, the PI3 kinase acts as an inhibitor of Best3 in vascular smooth muscle cells [60].

### Expression of *S. littoralis* Bestrophins

Our results reveal that SlitBest1a, SlitBest1b and SlitBest2 transcripts are co-expressed throughout the whole body, including cephalic, thoracic, abdominal regions and extending to the locomotor and chemosensory organs. These ubiquitous tissue distributions are in concordance with those described in other species, especially in *Xenopus laevis*, mouse and human where bestrophin isoforms are present in both cardiovascular, digestive, excretory, respiratory and nervous systems [27], [33], [35], [46]. Indeed it has been suggested that many physiological functions are linked to the activity of bestrophins in mammals. For instance, the human bestrophins are thought to be involved in secretory pathways of epithelial cells [16], [61], organelle trafficking [16], [30], retinal homeostasis [62] and cell volume regulation [44]. Therefore, it is highly probable that SlitBest1a, SlitBest1b and SlitBest2 exert a pleiotropic role in *S. littoralis* adults.

Interestingly, we found a predominant expression of SlitBest1a, SlitBest1b and SlitBest2 transcripts in *S. littoralis* proboscis and antennae, thus suggesting a major role of the encoded proteins in the functioning of the chemosensory system. To gain information on a putative role of SlitBest1a, SlitBest1b and SlitBest2 in the olfactory system, single cell RT-PCR experiments were conducted on cultured ORNs. Only the SlitBest1b transcript was detected in ORNs. Similar investigations in the mouse olfactory epithelium provided evidence for the expression of bestrophin 2 on the cilia of ORNs, the site of olfactory transduction [23].

Finally, we conducted a developmental analysis of SlitBest1b expression in male antennae. The amount of SlitBest1b transcript increases at the end of pupal stage and reaches a peak at the adult emergence which is maintained during the first days of adult life when moths initiate olfactory-dependent behaviors. The SlitBest1b expression during the developement is similar to that described for olfactory genes such as odorant binding proteins (OBPs) and ORs [63], [64] and might be associated to a massive synthesis of adult SlitBest1b required for the differentiation and/or the activity of ORNs.

### Expression of SlitBest1b in CHO cells Induces a Ca^2+^-dependent Anion Current

Expression of SlitBest1b in CHO cells induced CaC currents activated by physiological concentrations of cytosolic Ca^2+^, with an EC_50_ = 1.2 µM. This observation is consistent with previous findings that bestrophins induce CaC currents in heterologous expression [27], including insect bestrophins [28], [32].

The electrophysiological properties of the native CaC current in ORNs and of recombinant SlitBest1b exhibited similar properties. Both currents are anionic, have identical *I-V* relationships and present similar dependence on the Ca^2+^ concentration. Indeed, fitting the data of the relationship between the amplitude of CaC currents and the intracellular Ca^2+^ concentration yielded values of the same order of magnitude of EC_50_ (2.8 µM *vs.* 1.2 µM) and Hill coefficient (0.8 *vs.* 1.0) for the native CaC current in ORNs and the SlitBest1b currents, respectively. Both currents do not depend on the cell volume and have the same anion permeability sequence (I^−^>NO_3_^−^>Br^−^>Cl^−^>CH_3_SO_3_^−^>>gluconate^−^). Both currents develop slowly, partly inactivate over time (steady state current was 38±5% of peak current in ORNs and 58±6% of peak current for the SlitBest1b current), with comparable kinetics, have the same sequence of inhibitory efficiency of blockers (NPPB>flufenamic acid>niflumic acid), and present upon removal of blockers a transient peak current.

There is now compelling evidence demonstrating that several anoctamins (Ano) encode CaC channels [65]. In vertebrate ORNs, the CaC current is encoded by Ano2 and not by a bestrophin [22], [24]. The role of Best2, which is also expressed at the site of olfactory transduction in vertebrate ORNs, remains elusive. Unlike anoctamin-induced currents that in general exhibit an outward rectification [66], both CaC currents of *S. littoralis* ORNs [15] and recombinant SlitBest1b currents (this work) exhibited an almost linear *I-V* relationship. This is true also for native CaC currents of *Drosophila* S2 cells that were abolished by RNAi constructs to dBest1 and dBest2 [28]. Noteworthy is the exception of the recombinant *Drosophila* Best1 current in HEK cells that was outwardly rectified [32].

Bestrophins usually have a 10-times higher affinity for Ca^2+^ than do anoctamins [41]. The EC_50_ of CaC currents in *S. littoralis* ORNs and recombinant SlitBest1b currents are larger than the usual bestrophin EC_50_ [23], [33], [67]. However, important inactivation of both the native and recombinant currents most probably led us to underestimate peak currents and thus EC_50_.

### Role of SlitBest1b in ORNs

The expression of SlitBest1b in CHO-K1 cells induced a CaC current with electrophysiological properties similar to the CaC current recorded from moth ORNs. The most parsimonious explanation is that SlitBest1b encodes a CaC channel, strengthening previous convincing evidence that bestrophins form CaC channels when expressed in heterologous systems [66], [68]. Suggestions that bestrophins function as CaC channels were confirmed by the findings that mutations of specific amino acids in the predicted pore channel alter some biophysical properties of the protein (e.g. activation, conductance, ion selectivity, rectification) [28], [30], [35], [37], [67], [69].

Behavioral and electrophysiological experiments with pulsed stimuli revealed that moths are remarkably well adapted to the rapid changes in stimulus concentration they encounter in a natural odor plume. We previously identified a CaC current that likely contributes to the termination of insect ORN responses and thus participates to temporal coding in these sensory neurons. The present work shows for the first time that insect ORNs express a bestrophin transcript that is a good CaC channel candidate involved in olfactory transduction. Future studies should examine in more details whether bestrophin proteins contribute to insect olfactory coding, in particular to temporal coding.

However, we cannot exclude that SlitBest1b serve some other function than forming a CaC channel. Vertebrate bestrophins can be activated by cell swelling in the absence of Ca^2+^, indicating that they may be cell volume regulators [44], [45]. This is not the case for SlitBest1b whose activation did not depend on cell volume. Bestrophins can also regulate voltage-gated Ca^2+^ channels [39], [40] and it was suggested that bestrophins are regulators of Ca^2+^ channels rather than *bona fide* CaC channels [17], [27], [41], [61], [68]. The heterologous expression of SlitBest1b could potentially modify the expression, trafficking to the plasma membrane and/or function of endogenous CHO-K1 proteins and also of ORNs. In mice, Best2 appears to support growth and function of sensory cilia and might thus be involved in neurogenesis [47]. SlitBest1b expression was observed before the adult emergence and declined after 3 days of adult life by about 50% of the level measured at the adult emergence. Therefore, it might participate in the development of adult antenna during the pupal stage. Finally, it is possible to speculate that SlitBest1b contributes to setting the concentration of Cl^−^ or other anions (e.g. HCO_3_^-^ for which bestrophins are highly permeable [70]) in the sensillar lymph. The CaC channel hypothesis and the Ca^2+^ channel regulator hypothesis or role in antennal development for bestrophin are not mutually exclusive and bestrophins could act as multi-functional proteins [27]. Further studies will be required to determine the physiological role of SlitBest1b in ORNs.
